# The DmsABC Sulfoxide Reductase Supports Virulence in Non-typeable *Haemophilus influenzae*

**DOI:** 10.3389/fmicb.2021.686833

**Published:** 2021-07-22

**Authors:** Rabeb Dhouib, Marufa Nasreen, Dk Seti Maimonah Pg Othman, Daniel Ellis, Simon Lee, Ama-Tawiah Essilfie, Philip M. Hansbro, Alastair G. McEwan, Ulrike Kappler

**Affiliations:** ^1^School of Chemistry and Molecular Biosciences, Australian Infectious Disease Research Centre, The University of Queensland, St Lucia, QLD, Australia; ^2^QIMR Berghofer Medical Research Institute, Herston, QLD, Australia; ^3^Centre for Inflammation, Centenary Institute, School of Life Sciences, Faculty of Science, University of Technology Sydney, Sydney, NSW, Australia

**Keywords:** energy generation, *Haemophilus influenzae*, anaerobic respiration, sulfoxide, molybdenum enzyme, respiratory pathogen, sulfoxide reductase

## Abstract

Although molybdenum-containing enzymes are well-established as having a key role in bacterial respiration, it is increasingly recognized that some may also support bacterial virulence. Here, we show that DmsABC, a putative dimethylsulfoxide (DMSO) reductase, is required for fitness of the respiratory pathogen *Haemophilus influenzae* (Hi) in different models of infection. Expression of the *dmsABC* operon increased with decreasing oxygen availability, but despite this, a Hi2019^Δ*d*^*^*msA*^* strain did not show any defects in anaerobic growth on chemically defined medium (CDM), and viability was also unaffected. Although Hi2019^Δ*d*^*^*msA*^* exhibited increased biofilm formation *in vitro* and greater resistance to hypochlorite killing compared to the isogenic wild-type strain, its survival in contact with primary human neutrophils, in infections of cultured tissue cells, or in a mouse model of lung infection was reduced compared to Hi2019^WT^. The tissue cell infection model revealed a two-fold decrease in intracellular survival, while in the mouse model of lung infection Hi2019^Δ*d*^*^*msA*^* was strongly attenuated and below detection levels at 48 h post-inoculation. While Hi2019^WT^ was recovered in approximately equal numbers from bronchoalveolar lavage fluid (BALF) and lung tissue, survival of Hi2019^Δ*d*^*^*msA*^* was reduced in lung tissue compared to BALF samples, indicating that Hi2019^Δ*d*^*^*msA*^* had reduced access to or survival in the intracellular niche. Our data clearly indicate for the first time a role for DmsABC in *H. influenzae* infection and that the conditions under which DmsABC is required in this bacterium are closely linked to interactions with the host.

## Introduction

One of the most common types of biomolecular damage caused by oxidative stress is the oxidation of sulfur compounds including amino acids. Both cysteine and methionine are highly susceptible to oxidative damage, and there is increasing evidence that both thiol-based and molybdenum-containing methionine sulfoxide reductases protect bacterial pathogens from host-induced oxidative stress and thus support bacterial virulence (Ezraty et al., 2005, 2017; Denkel et al., 2013; Nasreen et al., 2020; Zhong et al., 2020).

We have previously characterized two methionine sulfoxide reductases from the human-adapted respiratory pathogen *Haemophilus influenzae* (Dhouib et al., 2016; Nasreen et al., 2020), a human pathobiont that asymptomatically colonizes and persists in the human nasopharynx. *H. influenzae* can migrate to other niches in the upper and lower respiratory tracts where it can cause both acute diseases such as otitis media, sinusitis, and pneumonia or exacerbate existing chronic lung conditions such as chronic obstructive pulmonary disease (COPD), bronchiectasis, asthma, and cystic fibrosis (Eldere et al., 2014; King and Sharma, 2015).

The two *H. influenzae* methionine sulfoxide reductases, MtsZ and MsrAB, are both part of the bacterium’s periplasmic defenses against oxidative stress (Dhouib et al., 2016; Nasreen et al., 2020). MsrAB is a peptide-sulfoxide reductase that can repair oxidative damage to *H. influenzae* cell envelope proteins using thiol redox chemistry. MsrAB is required for resistance to hypochlorite and was also shown to modulate host responses to *H. influenzae* infection (Nasreen et al., 2020). The protein-repairing activity of MsrAB is complemented by the activity of the molybdenum-containing MtsZ methionine sulfoxide reductase that reduces free methionine sulfoxide, thereby increasing the bioavailability of methionine for uptake by bacteria (Dhouib et al., 2016). MtsZ interacts with a membrane-bound cytochrome subunit, MtsY, and is connected to the *H. influenzae* respiratory chain as an alternative terminal reductase (Dhouib et al., 2016). In keeping with this, MtsZ has been shown to have a role in redox balancing.

MtsZ is one of two molybdenum-containing S-/N-oxide reductases found in *H. influenzae*, and while MtsZ is found in about 80% of *H. influenzae* strains, the DmsABC S-oxide reductase is completely conserved in *H. influenzae* strains (Othman et al., 2014; Dhouib et al., 2016). The *H. influenzae* DmsABC enzyme shows significant homology to its *E. coli* counterpart (∼74% amino acid sequence identity), which has been proposed to support *E. coli* growth *via* anaerobic respiration with dimethylsulfoxide (DMSO) as the electron acceptor (Sambasivarao and Weiner, 1991; Weiner et al., 1992). DmsA is the molybdenum-containing catalytic subunit and is located in the bacterial periplasm, while DmsB and DmsC are involved in electron transfer and anchoring the enzyme to the cell membrane, respectively. Although measurement of kinetic properties of the purified enzyme and *in vitro* growth experiments identified *E. coli* DmsABC as a DMSO reductase, the biological function of these enzymes requires reevaluation in the context of substrate availability. When considering *H. influenzae* DmsABC, it is important to note that DMSO is not present in the human respiratory tract, which is the only known niche of this pathobiont. Such considerations recently led us to demonstrate that *H. influenzae torZ*, which was thought to encode a trimethylamine-N-oxide reductase, actually encodes a methionine sulfoxide reductase, leading to the renaming of this gene as *mtsZ* (Dhouib et al., 2016).

The role of DmsABC in bacterial pathogens is mostly unstudied, although it has been observed that a strain of *Actinobacillus pleuropneumoniae* that lacked the *dmsABC* genes was avirulent in a pig model of lung infection; however, no mechanistic details were revealed (Baltes et al., 2003). As *A. pleuropneumoniae*, like *H. influenzae*, is a member of the Pasteurellaceae group of respiratory pathogens, we hypothesized that DmsABC might have a similar, key role in infection in *H. influenzae* and have used a combination of physiological and infection assays to test this hypothesis.

## Materials and Methods

### Growth of Bacterial Strains

Hi2019 wild-type (Hi2019^WT^) (Campagnari et al., 1987) and derivatives of this strain were cultivated on supplemented brain heart infusion (sBHI) (Johnston, 2010) or chemically defined growth medium (CDM) (Coleman et al., 2003). Both media (broth and agar forms) were supplemented with NAD and hemin which were added to a final concentration of 10 mg/L each. *E. coli* DH5α (Life Technologies) was grown in Luria Bertani (LB) broth or on LB agar (Sambrook et al., 1989) at 37°C. Where required, kanamycin (kan) (100 μg/ml *E. coli*; 20 μg/ml *H. influenzae*), spectinomycin (spec) (50 μg/ml *E. coli*; 20 μg/ml *H. influenzae*), and ampicillin (amp) (100 μg/ml *E. coli*) were added to culture media. For growth experiments, a microtiter plate reader CLARIOstar^®^ (BMG Labtech, Ortenberg, Germany) equipped with an Atmospheric Control Unit was used to monitor the growth of Hi2019 strains on CDM medium under aerobic (no controlled environment in the microplate reader chamber, shaking at 200 rpm), microaerobic (2.8% O_2_, shaking at 200 rpm), and anaerobic (5% CO_2_ with shaking for 30 s at 200 rpm just before reading) conditions. OD_600nm_ was measured every 7 min under aerobic and microaerobic conditions and every 30 min under anaerobic conditions for 24 h.

### Construction and Complementation of a HI2019^Δ^*^*dmsA*^* Strain

A DNA fragment (∼2,400 bp) covering the *dmsA* gene was amplified from Hi2019 genomic DNA using primers HI_dmsAfwd and HI_dmsArev (Supplementary Table 1) and cloned into pGEM^®^-T Easy (Promega, Madison, WI, United States) to create pGEM-*dmsA*. The kanamycin (kan) resistance cassette was amplified from the pUC-4K plasmid (Vieira and Messing, 1982) using primers pUC4K-PCR-F and pUC4K-PCR-R (Supplementary Table 1) and then inserted into the pGEM-*dmsA Bam*HI site yielding pGEM-*dmsA::kan*. This plasmid was linearized using *NcoI* and transformed into competent Hi2019 using the method described in Poje and Redfield (2003). In brief, Hi cultures were grown on sBHI to an OD_600nm_ of 0.25, harvested, and washed twice in MIV solution (Poje and Redfield, 2003) before resuspension in MIV and incubation at 37°C with shaking for 100 min to develop competence. One microgram of linearized plasmid was added to 1 ml of competent cells and incubated at 37°C for 30 min before plating on selective media. Transformants were selected on sBHI 20 μg/ml kan agar plates. The inactivation of *dmsA*, generating Hi2019^Δ^*^*dmsA*^*, was confirmed by PCR. MIV solution was prepared by combining 10 ml solution 21 (per liter: 4 g L-aspartate, 0.2 g L-glutamate, 1 g fumarate, 4.7 g NaCl, 0.87 g K_2_HPO_4_, 0.76 g KH_2_PO_4_, 0.2 ml Tween 80, pH 7.0) with 0.1 ml solution 22 (4 mg L-cytosine, 10 mg L-tyrosine dissolved in 1 ml 1 M HCl at 37°C followed by addition of 9 ml H_2_O and 6 mg L-citrulline, 20 mg L-phenylalanine, 30 mg L-serine, 20 mg L-alanine), 0.1 ml 0.1 M CaCl_2_, 0.1 ml 0.05 M MgCl_2_, and 0.1 ml 5% Difco vitamin-free Casamino acids.

To complement the HI2019^Δ^*^*dmsA*^* mutant, the *dmsABCDE* gene region (6,000 bp) was amplified using primers Hi2019dmsAcomp_Xma_F and Hi2019dmsAcomp_Xma_R (Supplementary Table 1) and cloned into p601.1-Sp2 (Johnston et al., 2007) using the *Xma*I site. The plasmid was linearized using *Bam*HI and transformed as described above, and Hi2019^Δ^*^*dmsA*^*^_*c*^ selected on sBHI plates containing 20 μg/ml kanamycin and 20 μg/ml spectinomycin. Correct integration of the construct was confirmed by PCR.

### Biofilm Quantification Assays

Biofilm biomass quantification and determination of colony-forming units in the biofilm were conducted as described (Dhouib et al., 2016). Briefly, non-typeable (NT) *H. influenzae* strains grown to an OD_600nm_ of 0.2–0.3 under anaerobic or microaerobic conditions in sBHI at 37°C were diluted to an OD_600nm_ of 0.05 before being distributed into 96-well microtiter plates (100 μl per well, supplier: Techno Plas, cat no. SMPSL) and incubated (24 h, 37°C) with or without shaking. After removing planktonic cells by washing the wells carefully with water, bound cells were stained using crystal violet as described in Schembri and Klemm (2001), allowing the quantification of biofilm formation. For quantification of colony-forming units present in the biofilm, planktonic cells were removed by washing with sterile water, and bound cells were incubated for 20 min with 200 μl of proteinase K (0.1 mg/ml in 1 × PBS) (Izano et al., 2009). The detached bacteria were mixed thoroughly by vigorous pipetting, serially diluted in 1 × PBS, and plated on sBHI agar to estimate the number of colony-forming units (CFU) per well. Biofilm analyses used three biological replicates for each strain, with several technical replicates for each biological replicate.

### Hypochlorite Susceptibility Assay

HOCl susceptibility assays were carried out as described in Dhouib et al. (2016) with slight modifications. Hi2019^WT^ and derivative strains were grown overnight on sBHI agar plates; the cell material was harvested using an inoculation loop and resuspended in 1 × PBS to an OD_600_ of 1.1. To 1.8 ml cell suspension in a 10-mL tube, 0.2 ml of water or freshly prepared 10 × HOCl stock solutions were added (final OD = 1.0), and samples incubated at room temperature with shaking for 60 min prior to serial dilution and determination of CFU/ml as described above. Final HOCl concentrations in samples ranged from 0.05 to 0.5 mM. Assays used three biological replicates with three technical replicates for each data point on each day the experiment was carried out and were repeated twice on independent days.

### General Molecular and Biochemical Methods

Standard methods were used throughout (Ausubel et al., 2005). All chemicals were purchased in analytical grade unless otherwise indicated. Competent *E. coli* were prepared as described in Hanahan et al. (1991). Plasmid and PCR product purification used the PureLink Plasmid DNA Miniprep and PCR Purification Kits (Life Technologies). Genomic DNA was isolated using the Genomic DNA Mini Kit (Life Technologies). Restriction enzymes were from Thermo Fisher (Waltham, MA, United States); T4 Ligase and RNAse inhibitor were from Promega. General PCR used GoTaq Master Mix Green (Promega), and high-fidelity amplification used Phusion Master Mix (Finnzymes/Thermo Fisher). Protein concentrations were determined using a bicinchoninic acid-based assay (BCA-1 kit, Sigma-Aldrich, St. Louis, MO, United States). Small-scale cell extracts of *H. influenzae* were prepared using BugBuster Master Mix (Novagen) as per the manufacturer’s instructions.

### Enzyme Assays

Sulfoxide activities in crude extracts from anaerobically grown cells were assayed as described in Dhouib et al. (2016). DMSO (17 mM), DL-methionine sulfoxide (10 mM), S-biotin sulfoxide (5 mM), and trimethylamine-N-oxide (TMAO) (20 mM) were used as electron acceptors. Specific enzyme activities are expressed as units (μmol substrate reduced per min) per mg protein and calculated as in Dhouib et al. (2016). Enzyme assays were repeated at least twice on different days using freshly prepared cell lysates, on each day at least three repeat assays were carried out.

### RNA Isolation and Quantitative RT-PCR (qRT-PCR) Analysis

RNA isolation used samples of liquid NTHi cultures at mid-exponential growth phase, the Illustra RNAspin Mini Kit (Cytiva, Marlborough, MA, United States) and RNAprotect Bacteria Reagent (Qiagen, Hilden, Germany). gDNA was removed from isolated RNA using the Turbo DNA-free^TM^ Kit (Life Technologies) and isolated RNA quantified using the Quant-IT RNA Kit (Life Technologies). PCR reactions using purified RNA as template were used to document successful removal of gDNA. cDNA was synthesized from 500 ng of RNA using SuperScript IV (Life Technologies) and random hexamer primers (Life Technologies). qRT-PCR was performed as described in Dhouib et al. (2016) and Othman et al. (2014). qRT-PCR reactions (10 μl) used diluted cDNA (1:100) as template, SYBR Green Master Mix (Applied Biosystems, Foster City, CA, United States), and primers (amplicon size: 120 bp) described previously in Othman et al. (2014) (Supplementary Table 1). Normalization used 16S gene expression and was performed as in Kappler et al. (2005) using Eq. 1, where PCReff = PCR efficiency, CT = cycle threshold, TG = target gene, and RG = reference gene.

(1) PCReffT⁢G-CTT⁢G/PCReffR⁢G-CTR⁢G

PCR efficiencies for the different genes were determined using LinReg (Ramakers et al., 2003) and were between 1.85 and 1.92. Co-transcription assays used cDNA prepared using gDNA-free RNA from anaerobic Hi2019 cultures in standard PCR reactions. To test co-transcription, the intergenic regions between adjacent genes were amplified using primer pairs listed in Supplementary Table 1.

### Tissue Culture Infection

Human bronchial epithelial 16HBE14 cells (Gruenert et al., 1988), kindly provided by Dr. Kirsten Spann (Queensland University of Technology), were seeded in Minimal Essential Medium (MEM) supplemented with 10% fetal bovine serum (sMEM) at an approximate density of 2 ^∗^ 10^5^ cells/ml into 24-well culture dishes (Greiner Bio-One, Kremsmünster, Austria) (1 ml/well) for adherence and invasion assays or into 175-cm^2^ flasks (Corning, Tewksbury, MA, United States) (40 ml/flask) for maintenance. Confluent 16HBE14 monolayers were washed once with prewarmed sMEM and then infected with Hi2019*^WT^*, Hi2019^Δ^*^*dmsA*^*, or Hi2019^Δ^*^*dmsA*^*^_*c*^ diluted in sMEM to 2 ^∗^ 10^7^ bacteria/ml, giving a multiplicity of infection (MOI) of 1:100 (epithelial cells: bacteria). Bacterial adherence and invasion were determined as described previously (Dhouib et al., 2015, 2016). Determination of intracellular bacteria used a standard gentamicin-protection assay (St Geme and Falkow, 1990). In brief, infected cells were washed to removed planktonic and loosely adherent bacteria and incubated for 1 h in sMEM containing 50 μg/ml gentamicin followed by saponin lysis and serial dilution for determination of bacterial CFU present. Assays used at least three biological replicates; replicate experiments were carried out on different days.

### Neutrophil Killing Assays

Assays were carried out as in Dhouib et al. (2015) under UQ human ethics approval 2010000491. Human neutrophils were isolated and purified from venous blood using the PolyMorphPrep kit (Axis Shield, Dundee, United Kingdom) as per the manufacturer’s instructions and seeded into 96-well plates at 2 ^∗^ 10^5^ cells/well. *H. influenzae* strains grown overnight on fresh sBHI-agar plates were resuspended in RPMI medium containing 2% heat-inactivated autologous human plasma, diluted to 2 ^∗^ 10^7^ CFU/ml in the same medium, and then added to neutrophils at an MOI of 1:10 (neutrophils: bacteria) (Walker et al., 2007). Plates were centrifuged at 500 × *g* for 10 min then incubated at 37°C with 5% CO_2_ for 30 min. After incubation, the remaining neutrophils were lysed with water, the content of each well serially diluted in BHI and plated on sBHI-agar for overnight incubation and enumeration of CFU. Experiments were repeated twice with three technical replicates for each determination.

### Immunofluorescence Microscopy

16HBE14 cells were grown to confluence on glass coverslips (13 mm, #1, ProSciTech, Kirwan, QLD, Australia), placed in 24-well plates, and then infected with NTHi strains as described for adherence and invasion assays. Planktonic cells were removed by washing three times with 1 × PBS after 4 or 24 h of incubation at 37°C with 5% CO_2_. Epithelial cells and bacteria were then fixed in 4% paraformaldehyde for 15 min, permeabilized with 0.1% Triton X-100 in 1 × PBS for 5 min, and blocked overnight at 4°C with blocking buffer (2% BSA, 0.02% sodium azide in 1 × PBS) for immunofluorescence staining (Hogardt et al., 2000).

Immunofluorescence staining of NTHi was performed as described in Dhouib et al. (2016). Briefly, NTHi were labeled using the primary antibody 6E4 (200 μl of 1:100 dilution) kindly provided by Prof. Michael Jennings [Institute for Glycomics, Griffith University, Australia; (Erwin et al., 2006)] for 3 h at room temperature. Coverslips were washed three times with blocking buffer (500 μl) and then incubated for 2 h in the dark with secondary anti-mouse IgG (whole molecule)-FITC antibody produced in goat (Sigma-Aldrich) (200 μl of 1:100 dilution). Epithelial cells were stained with CellTracker^TM^ Orange CMTMR fluorescent dye (Thermo Fisher; 200 μl of 1 μg/ml solution) for 1 h at room temperature in the dark before coverslips were mounted onto slides using ProLong^®^ Gold Antifade Reagent (Thermo Fisher). Immunofluorescence images were acquired using an Axiophot 2 epifluorescence light microscope (Zeiss, Jena, Germany).

### Mouse Model of Lung Infection

Experimental animal procedures were carried out in strict accordance with the recommendations of the NSW Animal Research Regulation 2005 and the Australian code of practice for the care and use of animals for scientific purposes of the National Health. Protocols were approved by the Animal Care and Ethics Committees of the University of Newcastle and the University of Queensland (UN/SCMB/335/13/NHMRC). For Hi pulmonary infection, a mouse model described previously by Dhouib et al. (2016) and Morey et al. (2013) was used. Hi strains were grown in sBHI for 16 h at 37°C with 5% CO_2_. BALB/c female mice (5 to 6 weeks old) were inoculated intranasally with 30 μl of a bacterial suspension containing 10^7^ CFUs. Groups of six mice were euthanized and necropsied at 0, 24, 48, and 72 h. Bacterial recovery was determined as in Dhouib et al. (2016); Essilfie et al. (2011), Essilfie et al. (2012), and Essilfie et al. (2015).

### Statistical Analyses

Statistical testing was carried out using the Prism 9 software package. Depending on the data structure, either *t*-tests, one-way ANOVA, or two-way ANOVA was used as detailed in the figure legends. A *p* value < 0.05 was considered statistically significant.

## Results

### DmsABC Expression in *H. influenzae* Is Associated With Anaerobiosis but Shows No Substrate-Dependent Induction

**FIGURE 1:**
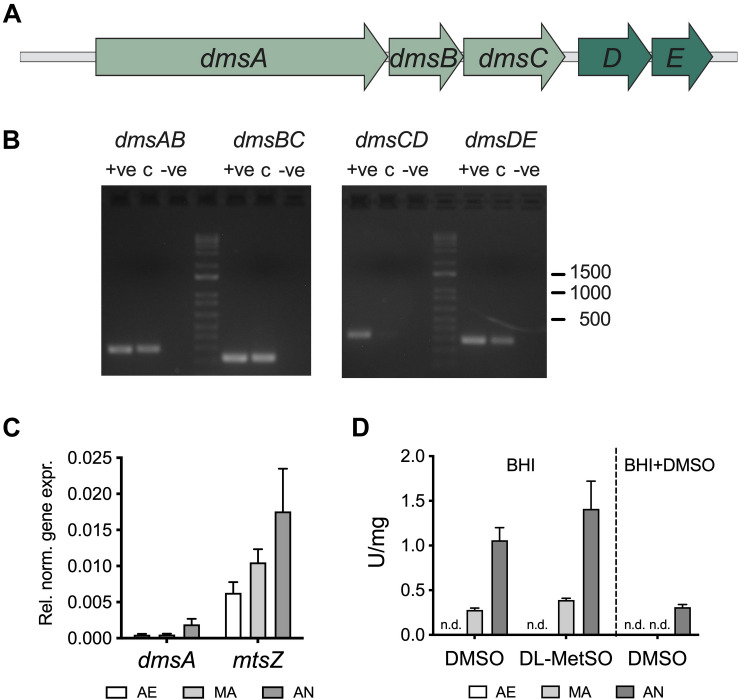
Gene expression and biochemical activity of S-oxide reductases in *Haemophilus influenzae*. **(A)** Schematic representation of the *H. influenzae dms* operon **(B)** Co-transcription of *dmsABCDE* genes. Intergenic regions of adjacent genes were amplified using cDNA from Hi2019 cultures grown under anaerobic conditions as the template. +ve – positive control (gDNA), c – cDNA, –ve – negative control. **(C)** Expression of the *dmsA* and *mtsZ* genes in *H. influenzae* as a function of oxygen availability. Data are derived from qRT-PCR using RNA from Hi2019 cultures growing on CDM medium under aerobic (AE), microaerobic (MA), or anaerobic (AN) conditions; gene expression was normalized to 16S. **(D)** S-oxide reductase activity in cell extracts from Hi2019 cultures grown on BHI medium with or without DMSO added under AE, MA, or AN conditions. N.d. – not detected. The enzyme activity pattern matches the gene expression patterns and also reveals that DMSO reduces the expression of S-oxide reductases in *H. influenzae.*

In *H. influenzae*, the DmsABC sulfoxide reductase is encoded by a five-gene operon, *dmsABCDE* (Hi2019: C645_RS06125–C645_RS06105) (Figure 1A), that is completely conserved in genomes of *H. influenzae* strains. The *dmsABC* genes encode the structural components of the enzyme, namely, the catalytic subunit DmsA, an iron-sulfur cluster-containing electron transfer subunit, DmsB, and the membrane anchor subunit, DmsC. The *dmsDE* genes encode two chaperones, one of which, DmsD, is essential for DmsABC maturation in *E. coli*, even though in *E. coli dmsD* is part of the *ynf* operon that encodes a DmsABC-related enzyme (Ray et al., 2003). The gene we have designated *dmsE* encodes a protein homologous to the iron-sulfur cluster protein NapF from *E. coli* that is required for maturation of the Nap-type nitrate reductase, another periplasmic molybdenum-containing enzyme (Olmo-Mira et al., 2004).

The *dmsDE* genes are separated from *dmsABC* by 111 bp, while all other genes are separated by 2 to 10 bp. Co-transcription analysis indicated that two major transcriptional units are present, *dmsABC* and *dmsDE* (Figure 1B). Gene expression analysis for the non-typeable COPD isolate Hi2019 revealed that *dmsA* is induced under anaerobic conditions (Figure 1C), which is in keeping with *dmsA* being controlled by the fumarate-nitrate regulator (FNR) in both *E. coli* and *H. influenzae* (McNicholas et al., 1997; Constantinidou et al., 2006; Harrington et al., 2009). A similar gene expression pattern was previously identified by us for the *H. influenzae* reference strain RdKW20 (Othman et al., 2014).

We also assessed levels of S-oxide reductase activity in Hi2019 cell extracts. As both MtsZ and DmsABC use viologen dyes as electron donors in *in vitro* enzyme assays, S-oxide reductase activity in *H. influenzae* crude extracts is the result of the combined activity of MtsZ (Dhouib et al., 2016) and DmsABC. Enzyme assays revealed a reduction in DMSO reductase activity in cell extracts from cultures grown on DMSO-containing media for both the Hi2019 wild-type strain and a Hi2019^Δ^*^*mtsZ*^* gene knockout strain where DmsABC is the only S-oxide reductase present (Figure 1D and Supplementary Figure 1). This suggests that in *H. influenzae* neither the MtsZ nor the DmsABC sulfoxide reductases are induced in the presence of DMSO, which is similar to the situation in *E. coli* (McNicholas et al., 1997; Constantinidou et al., 2006; Harrington et al., 2009).

### Under *in vitro* Conditions, DmsABC Is Not Required for *H. influenzae* Viability

To better understand the contribution of DmsABC to *H. influenzae* physiology, we then used the non-typeable *H. influenzae* (NTHi) strain Hi2019 to construct a Hi2019^Δ^*^*dmsA*^* mutant as well as a strain complemented for the mutation, Hi2019^Δ^*^*dmsAc*^*. Both strains were verified using PCR, and enzyme assays were used to determine the effect of the mutation on sulfoxide reductase activity.

**FIGURE 2:**
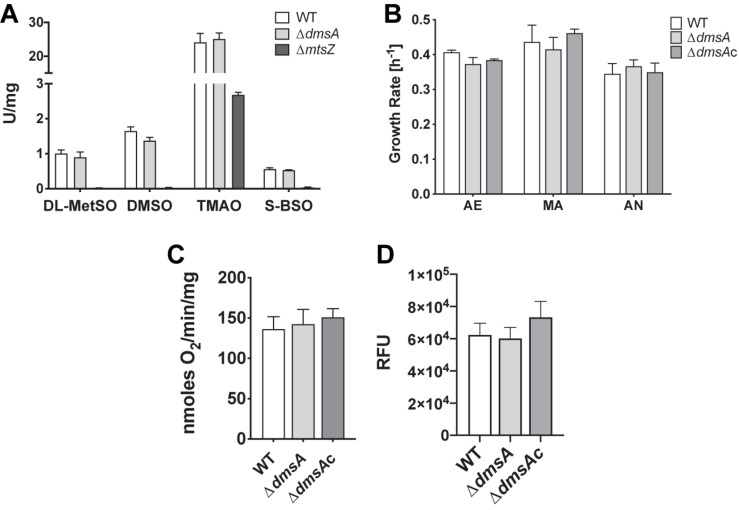
Comparison of *in vitro* physiological properties of Hi2019^WT^ and Hi2019^Δ^*^*dmsA*^*. **(A)** S- and N-oxide reductase activities in Hi2019^Δ^*^*dmsA*^* and Hi2019^Δ^*^*mtsZ*^* strains using known S- and N-oxide reductase substrates: DL-MetSO – DL-methionine sulfoxide; DMSO – dimethylsulfoxide, TMAO – trimethylamine N-oxide, S-BSO – S biotin sulfoxide. **(B)** Growth rate of Hi2019^WT^, Hi2019^Δ^*^*dmsA*^*, and Hi2019^Δ^*^*dmsAc*^* on CDM medium with glucose under aerobic (AE) microaerobic (MA) or anaerobic (AN) conditions. None of the data showed statistically significant differences (*p* < 0.05). **(C)** Oxygen-dependent respiration of Hi2019^WT^, Hi2019^Δ^*^*dmsA*^*, and Hi2019^Δ^*^*dmsAc*^* with glucose as the substrate. **(D)** Bacterial cell health of Hi2019^WT^, Hi2019^Δ^*^*dmsA*^*, and Hi2019^Δ^*^*dmsAc*^* following growth on CDM glucose medium determined using a BacTiter-Glo assay (Promega). RFU – relative fluorescence units. Statistical analyses **(B–D)** used one-way ANOVA (*post hoc* test: Dunnett), but returned no results with *p* < 0.05.

Enzyme assays using four known substrates converted by both the MtsZ and DmsABC sulfoxide reductases indicate that MtsZ is the major sulfoxide reductase in strain Hi2019, with mutation of *dmsA* only leading to a partial loss of MetSO reductase (12%) and DMSO reductase (17%) activity compared to the WT. Activity with S-biotin sulfoxide (BSO) and trimethylamine N-oxide (TMAO) as substrates was essentially unaffected (Figure 2A). For comparison, we included assays with a Hi2019 strain carrying a mutation in the *mtsZ* gene (Dhouib et al., 2016), and this clearly showed that MtsZ is the main enzyme responsible for S- and N-oxide reductase activity in Hi2019 *in vitro* (Figure 2A).

During infection, *H. influenzae* may encounter environments with different levels of oxygen availability, and based on the induction of *dmsABC* gene expression under anaerobic conditions, we expected that likely phenotypes associated with a loss of this enzyme would be most obvious under oxygen limitation, which is typically encountered during infection. We tested this by growing the strains on CDM with glucose in the presence of differing oxygen levels. However, contrary to expectations, no significant differences were observed between the WT, the *dmsA* mutant, and complemented strains (Figure 2B). Another potential phenotype associated with a loss of DmsABC could be a change in the ability to carry out oxygen-dependent respiration which we previously demonstrated to be reduced in a Hi2019^Δ^*^*mtsZ*^* strain (Dhouib et al., 2016). However, there was no change (Figure 2C), and similarly, no difference in cell viability following microaerobic growth was observed using a BacTiter-Glo assay that measures ATP content (Figure 2D). A large-scale investigation of metabolic and physiological changes in Hi2019^Δ^*^*dmsA*^* compared to Hi2019^WT^ using Omnilog Phenotypic microarrays was carried out and confirmed that the strain had no defects in growth in the presence of osmotic stressors, but showed some changes in nitrogen and phosphorous source utilization, where use of purine and pyrimidine nucleosides as nitrogen sources (e.g., inosine, cytidine) and nucleotides (different guanosine, uridine, and cytosine phosphates) as phosphorous sources was impaired (Supplementary Figure 2). While we previously observed a similar phenotype for mutations in the second *H. influenzae* sulfoxide reductase, MtsZ, the significance of this and how it could be linked to the loss of sulfoxide reductase activity is unclear at present. In summary, it would appear that DmsABC is not required for Hi2019 growth under *in vitro* conditions, even when using a CDM where nutrients resemble those found in the host.

### Hi2019^Δd*msA*^ Shows an Increase in Biofilm Formation and Increased Resistance to HOCl

**FIGURE 3:**
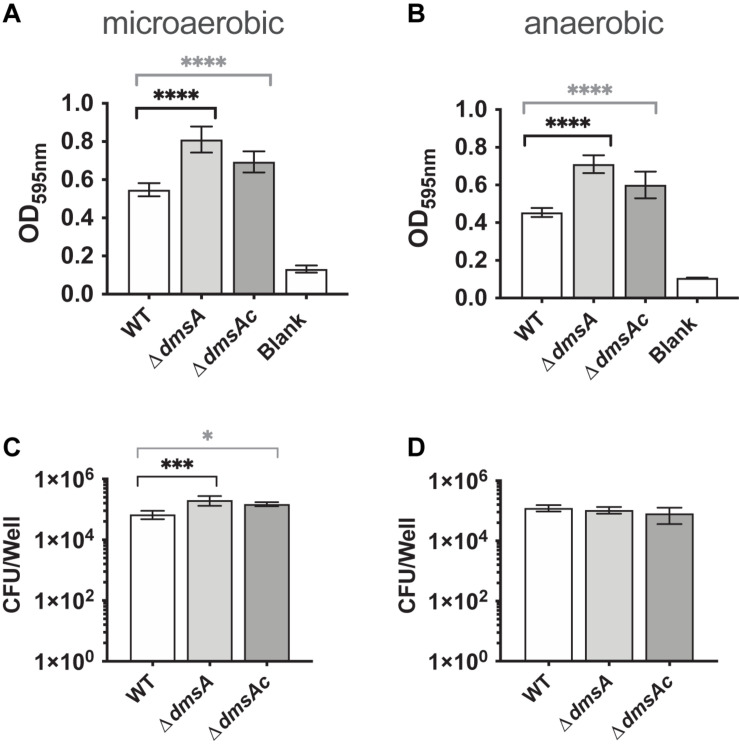
Biofilm formation **(A,B)** and survival **(C,D)** of Hi2019^WT^, Hi2019^Δ^*^*dmsA*^*, and Hi2019^Δ^*^*dmsAc*^* under microaerobic and anaerobic conditions. Biofilm formation was determined following staining with crystal violet. Culturable CFU present in the biofilm were determined following digestion of the biofilm matrix and serial dilution of vigorously resuspended bacteria. Statistical analyses used one-way ANOVA (*post hoc* test: Dunnett), ^∗^*p* < 0.05, ^∗∗∗^*p* < 0.001, ^****^p < 0.0001.

We then tested *H. influenzae* physiological traits relevant to infection such as biofilm formation. Unexpectedly, the Hi2019^Δ^*^*dmsA*^* strain showed increased biofilm formation under both microaerobic and anaerobic conditions, with 1.48- and 1.56-fold increases compared to Hi2019^WT^, while the Hi2019^Δ^*^*dmsAc*^* strain showed a 1.2-fold increase in biofilm formation (Figures 3A,B). This was associated with a 3.3-fold (*p* = 0.0004, one-way ANOVA) and 2.7-fold (*p* = 0.0193, one-way ANOVA) increase in bacteria present in biofilms of Hi2019^Δ^*^*dmsA*^* and Hi2019^Δ^*^*dmsAc*^*, respectively (Figures 3C,D). We assume that this modest change in biofilm formation and culturable bacteria present in the biofilm will have little or no biological significance. However, the increase in biofilm formation could contribute to increased resistance of the Hi2019^Δ^*^*dmsA*^* strain to adverse environmental conditions.

**FIGURE 4:**
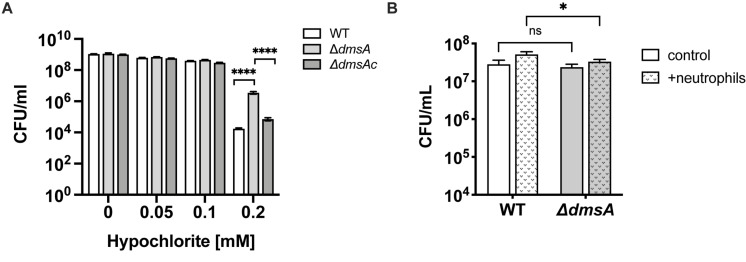
Sensitivity of Hi2019^Δ^*^*dmsA*^* to hypochlorite-induced stress. **(A)** survival of Hi2019^WT^ Hi2019^Δ^*^*dmsA*^* and Hi2019^Δ^*^*dmsAc*^* in the presence of increasing concentrations of hypochlorite. Bacteria were suspended in 1 × PBS prior to exposure to HOCl for 60 min. **(B)** Survival of Hi2019^WT^ and Hi2019^Δ^*^*dmsA*^* following exposure to human primary neutrophils for 30 min. Statistical testing for Panel **(A)** data used one-way ANOVA (*post hoc* test: Tukey–Kramer), and two-way ANOVA (*post hoc* test: Bonferroni) for data in Panel **(B)**; ^****^p < 0.0001, ^∗^*p* < 0.05, ns – not significant.

We tested this using exposure to HOCl, which we had also previously tested on a strain lacking the MtsZ enzyme where no change in resistance was observed (Dhouib et al., 2016). Interestingly, at a concentration of 0.2 mM HOCl that is partially bactericidal for NTHi, Hi2019^Δ^*^*dmsA*^* was two orders of magnitude more resistant to HOCl killing than Hi2019^WT^ (*p* < 0.0001, one-way ANOVA), while the complemented strains showed WT sensitivity to HOCl (Figure 4A). These results indicate that loss of DmsABC might influence virulence of *H. influenzae* by triggering changes in gene expression that lead to altered biofilm formation and resistance to HOCl, which is produced at sites of infection by the host enzyme myeloperoxidase (Rosen et al., 2009).

### Loss of DmsABC Reduces *H. influenzae* Fitness in Interactions With Human Bronchial Epithelial Cells and Neutrophils

**FIGURE 5:**
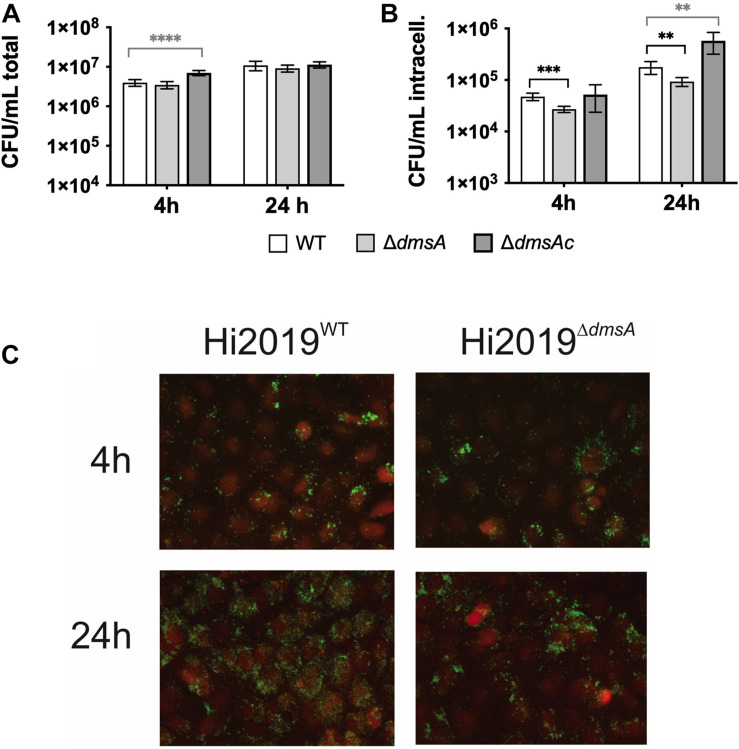
Interaction of *H. influenzae* strains with 16HBE14 human bronchial epithelial cells. **(A)** Numbers of total tissue cell adherent bacterial colony-forming units (CFU) 4 or 24 h post-infection with Hi2019^WT^ Hi2019^Δ^*^*dmsA*^* or Hi2019^Δ^*^*dmsAc*^*. **(B)** Bacterial intracellular CFU at 4 and 24 h post-infection. **(C)** Fluorescence microscopy of 16HBE14 tissue cells infected with Hi2019^WT^ or Hi2019^Δ^*^*dmsA*^*. 16HE14 cells are stained with CellTracker orange; Hi2019 appears in green following antibody-mediated detection. While at 4 h post-infection both strains show similar colonization patterns, after 24 h growth of Hi2019^Δ^*^*dmsA*^* shows a different pattern with less even colonization of the tissue cells. Images are representative of three independent experiments. Statistical testing **(A,B)** used unpaired, two-tailed *t*-tests, ^∗∗^*p* < 0.01, ^∗∗∗^*p* < 0.001, ^****^p < 0.0001.

Based on these considerations, we then tested the ability of Hi2019^Δ^*^*dmsA*^* to infect 16HBE14 human bronchial epithelial tissue cells as well as survival of this strain in the presence of primary human neutrophils. Confluent cultures of 16HBE14 were infected at an MOI of 100:1 with the different *H. influenzae* strains, and numbers of total adherent/cell-associated bacteria and internalized bacteria were determined after 4 and 24 h of infection. The Hi2019^Δ^*^*dmsA*^* strain showed a small reduction in total, tissue-cell adherent cell numbers (∼20% reduction, n.s, unpaired *t*-test) compared to Hi2019^WT^. However, both at 4 and 24 h, the numbers of internalized Hi2019^Δ^*^*dmsA*^* bacteria were reduced by a factor of 2 (4 h: *p* = 0.0002; 24 h: *p* = 0.003; unpaired *t*-test). This phenotype was reversed in Hi2019^Δ^*^*dmsAc*^* (Figures 5A,B). Epifluorescence microscopy of Hi2019-infected 16HBE14 cells documented the reduction of bacterial loads for infections with the Hi2019^Δ^*^*dmsA*^* strain after 24 h of incubation, while at 4 h post-infection essentially no difference was observed (Figure 5C).

As the Hi2019^Δ^*^*dmsA*^* strain showed increased resistance to HOCl killing, we also tested its survival in the presence of primary human neutrophils. NTHi WT strains are highly resistant to neutrophil-mediated killing, and numbers of culturable bacteria usually increase during the first 30 min of incubation (Dhouib et al., 2016). We observed a mild attenuation of this phenotype in Hi2019^Δ^*^*dmsA*^*, with culturable bacterial numbers of this strain increasing 1.4-fold compared to the control where no neutrophils were present, while for Hi2019^WT^ a 1.8-fold increase was observed (*p* = 0.0234, two-way ANOVA) (Figure 4B).

### DmsABC Is Required for Successful NTHi Lung Infection in Mice

**FIGURE 6:**
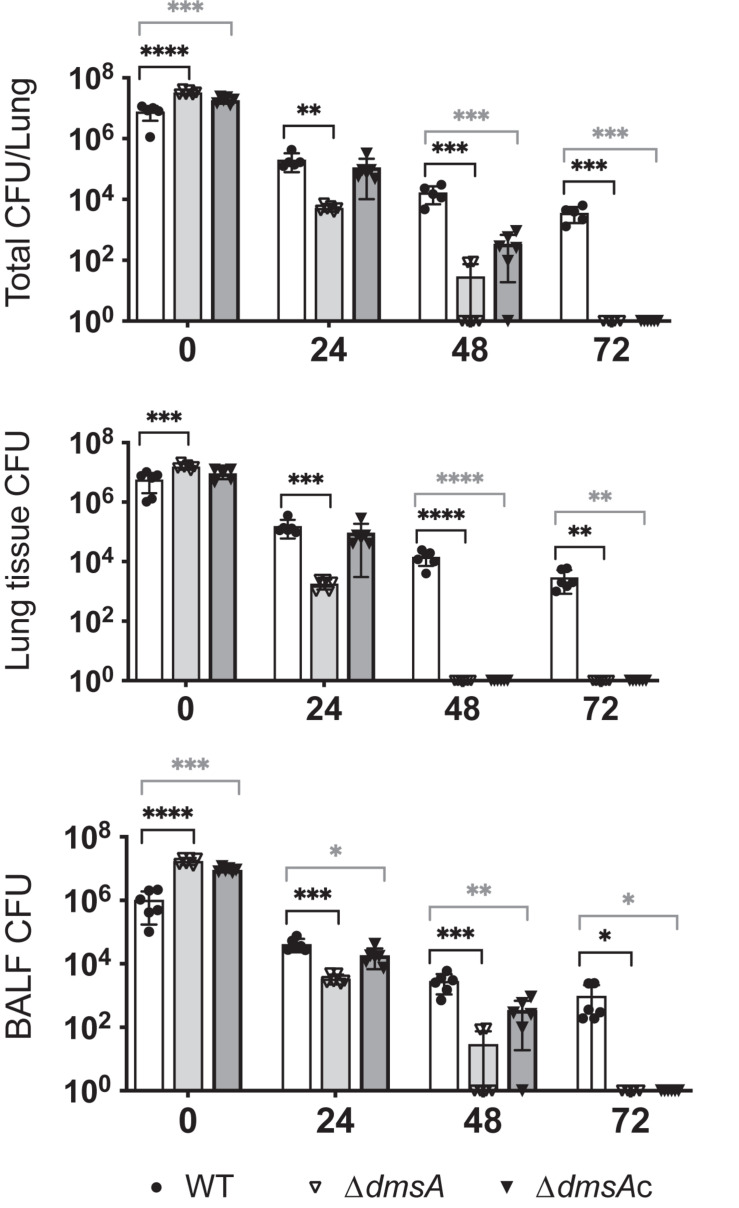
Survival of Hi2019^WT^ (white), Hi2019^Δ^*^*dmsA*^* (light gray), and Hi2019^Δ^*^*dmsAc*^* (gray) in a mouse model of lung infection. **Top:** Total CFU/lung (tissue + bronchoalveolar lavage fluid (BALF)), **Middle:** CFU/lung tissue, **Bottom:** CFU/BALF. Hi2019^Δ^*^*dmsA*^* is essentially cleared after 48 h of infection, an effect that was mostly reversed in Hi2019^Δ^*^*dmsAc*^*. Each group consisted of six mice. Statistical testing used one-way ANOVA (*post hoc* test: Dunnett), ^∗^*p* < 0.05, ^∗∗^*p* < 0.01, ^∗∗∗^*p* < 0.001, ^****^p < 0.0001.

Given that the only significant phenotypes of the Hi2019^Δ^*^*dmsA*^* strain were associated with interactions with host cells, we then tested the ability of this strain to survive in a mouse model of lung infection. Mice were infected with 10^7^ CFU of NTHi, and bacterial loads were tested every 24 h up to 72 h as in Dhouib et al. (2016) and López-López et al. (2020). As expected, CFU loads of Hi2019^WT^ decreased over time, with 3.6 ^∗^ 10^3^ CFU/lung remaining at 72 h. In contrast, Hi2019^Δ^*^*dmsA*^* showed a severe defect in survival, where bacteria were only detectable in all mice (*n* = 6) at 24 h post-infection (5.4 ^∗^ 10^3^ CFU/lung), while at 48 h bacteria were only detectable in two of six mice at levels of 85 and 90 CFU/lung (Figure 6). This defect was not completely reversed in the Hi2019^Δ^*^*dmsAc*^* strain, but it was significantly alleviated with the strain only dropping below the detection level at 72 h. Analysis of bacterial loads present in lung tissue as opposed to BALF showed that the Hi2019^Δ^*^*dmsA*^* phenotype was more pronounced for lung tissue samples, reinforcing that the survival defect likely is associated with a reduction in the ability of the strain to colonize and replicate intracellularly in host cells.

We also determined the number of immune cells in BALF of mice. Infection with Hi2019^WT^ led to an influx of immune cells into the mouse airways that peaked at 48 h; exposure to Hi2019^Δ^*^*dmsA*^* led to a delayed influx of immune cells after 24 h (one-way ANOVA, *p* < 0.0001) (Supplementary Figure 3A). This then increased after 48 h, until at 72 h post-infection immune cells were increased in Hi2019^Δ^*^*dmsA*^* infections compared to the WT (one-way ANOVA, *p* = 0.071). This was mostly driven by changes in both neutrophil and macrophage numbers (Supplementary Figures 3B,C) and was not observed for the strain in which the mutation had been complemented.

## Discussion

There is increasing evidence that the bacterial ability to repair damage to sulfur compounds, such as sulfoxide formation on biomolecules, is essential for virulence in a variety of pathogens including *Salmonella* and *Helicobacter* species (Alamuri and Maier, 2004, 2006; Denkel et al., 2013; Trivedi et al., 2015). Here, we provide the first evidence that contrary to its accepted role in supporting anaerobic respiration with DMSO, the DmsABC DMSO reductase from the respiratory pathogen *H. influenzae* is essential for bacterial survival during infection. The *H. influenzae* DmsABC S-oxide reductase shares extensive sequence homology with DmsABC from *E. coli*, which has been proposed to support anaerobic respiration of *E. coli* in the presence of DMSO (Weiner et al., 1992). Other similarities between the *H. influenzae* and *E. coli* DmsABC enzymes include that their expression is induced when oxygen availability is low, while the proposed substrate, DMSO, had no effect or even an inhibitory effect on S-oxide reductase activity (McNicholas et al., 1997). However, the human respiratory tract which is the only known niche for *H. influenzae* is devoid of this chemical, calling a similar function of *H. influenzae* DmsABC into question. Interestingly, while *H. influenzae* cell extracts contain significant amounts of S-oxide reductase activity, the majority could be attributed to *H. influenzae* MtsZ. This is a Mo-containing methionine sulfoxide reductase we characterized previously (Dhouib et al., 2016), but which, unlike the strictly conserved DmsABC, occurs only in approximately 80% of *H. influenzae* strains. The enzyme activity data matched the magnitude of expression observed for the *dmsA* and *mtsZ* genes, respectively (Figures 1C,D), and this seemed to indicate a minor role for DmsABC in *H. influenzae* physiology. *In vitro* characterization of a Hi2019^Δ^*^*dmsA*^* strain appeared to bear this out as no major phenotypes were discovered other than a slight increase in biofilm formation and, unexpectedly, increased resistance to HOCl killing.

While the increase in HOCl resistance suggested that the Hi2019^Δ^*^*dmsA*^* strain might have an increased resistance to killing by HOCl-producing phagocytes and might show increased virulence, assays using different models of infection including human bronchial cells, human primary neutrophils, and a mouse model of lung infection all revealed decreased Hi2019^Δ^*^*dmsA*^* fitness during infection.

The loss of DmsABC led to a reduction in invasion and/or survival in human bronchial cells, while exposure of the Hi2019^Δ^*^*dmsA*^* strain to primary human neutrophils reduced survival compared to the wild-type strain (Naylor et al., 2007; Dhouib et al., 2016). The most significant phenotype, however, was observed for the mouse model of lung infection, where the Hi2019^Δ^*^*dmsA*^* strain was essentially cleared 48 h post-infection, while Hi2019^WT^ was still present after 72 h. This survival defect was more pronounced for lung tissue than BALF, which contains surface-adherent bacteria and matches our observation of a reduction in intracellular bacteria for Hi2019^Δ^*^*dmsA*^* in the tissue culture model of infection (Figure 6). This reduction in survival was even more surprising as infection with Hi2019^Δ^*^*dmsA*^* led to a delay in the influx of neutrophils and macrophages into mouse lungs (Supplementary Figure 3). These results document the importance of DmsABC for *H. influenzae* virulence and are also consistent with the reported attenuation of virulence caused by a *dmsABC* mutation in *Actinobacillus pleuropneumoniae* in a pig model of pleuropneumonia (Baltes et al., 2003). Our attempts to understand the mechanism by which DmsABC supports virulence, however, have only managed to rule out defects in bacterial growth, respiration, and viability in the Hi2019^Δ^*^*dmsA*^* strain and show that the observed increases in biofilm formation and HOCl resistance are not sufficient to compensate for the loss of DmsABC during infection.

Several studies have used transposon mutagenesis to identify genes essential for *H. influenzae* virulence under different conditions, including two studies that investigated survival of *H. influenzae* transposon mutants in mouse models of lung infection in the presence or absence of a coinfection with influenza A virus. Following a 24-h selection time, no fitness defect associated with insertions in the *dmsABCDE* genes was detected in either study for *H. influenzae* strains encoding both DmsABC and the MtsZ methionine sulfoxide reductase (Gawronski et al., 2009; Wong et al., 2013). A likely explanation for the observed differences between our results and the transposon library selection experiments could be the short incubation time of 24 h used in the transposon studies (Gawronski et al., 2009; Wong et al., 2013). At 24 h post-infection, we observed only a small reduction in Hi2019^Δ^*^*dmsA*^* cell numbers, but a similarly small reduction in bacterial cell numbers carrying *dmsABCDE* mutations may not have been as apparent in the transposon library selection experiment. In our experiment, strong changes in survival of the Hi2019^Δ^*^*dmsA*^* strain were only apparent at 48 and 72 h post-inoculation.

Another possible explanation for some variation especially in short-term selection experiments may be linked to *dmsABCDE* being subject to epigenetic regulation by phase variation in *H. influenzae*. Two studies observed changes in the expression of several *dms* genes depending on whether the ModA2 methyltransferase that drives phase variation was active (“On”) or in an “Off” state (Atack et al., 2015; Brockman et al., 2018). However, even though both studies used *H. influenzae* strain 723, the results reported reveal a further complexity in the regulation of *dmsABC*, where in biofilms *dmsABCDE* expression increased with ModA2 in an “Off” state, while in a *H. influenzae* 723 ModA2ON mutant the expression of *dmsABCDE* was increased following growth in liquid BHI medium (Atack et al., 2015; Brockman et al., 2018).

While the mechanistic understanding of DmsABC function during lung infection remains elusive, it appears that this enzyme is likely not involved in converting methionine or biotin sulfoxide as its main substrate, as both activities were essentially abolished in the Hi2019^Δ^*^*mtsZ*^* strain. However, based on the physiology of this strain and the presence of a phenotype only in the *in vivo* infection models, it would appear that this enzyme likely converts a substrate that is present only during infection. We propose that this could be another S- or N-oxide that forms during infection of animal lung tissue, likely as a result of inflammation and the associated formation of reactive oxygen and nitrogen species. The exact nature of this compound remains unknown, and further work is required to identify it and to fully understand the role of DmsABC in the physiology and virulence of *H. influenzae*.

## Data Availability Statement

The original contributions presented in the study are included in the article/Supplementary Material, further inquiries can be directed to the corresponding author.

## Ethics Statement

The studies involving human participants were reviewed and approved by the University of Queensland Ethics Committee. The patients/participants provided their written informed consent to participate in this study. The animal study was reviewed and approved by the Animal Care and Ethics Committees of the University of Newcastle and The University of Queensland.

## Author Contributions

RD, MN, DSMPO, DE, SL, A-TE, and UK carried out the experimental work described in the manuscript. RD, A-TE, PH, AM, and UK were responsible for the conceptualization of experiments, UK, AM, A-TE, and PH acquired the funding. RD, MN, DSMPO, A-TE, and UK created the visualizations. UK and RD drafted the manuscript. All authors contributed to the data analysis and editing of the manuscript.

## Conflict of Interest

The authors declare that the research was conducted in the absence of any commercial or financial relationships that could be construed as a potential conflict of interest.
