# Supplementary material for: The DmsABC Sulfoxide Reductase Supports Virulence in Non-typeable Haemophilus influenzae
Source: Front Microbiol. 2021 Jul 22;12:686833. doi: 10.3389/fmicb.2021.686833 (PMC8340005; doi:10.3389/fmicb.2021.686833)
Supplement: Supplementary file 1 [file Data_Sheet_1.docx]

The DmsABC sulfoxide reductase is essential for virulence in *Haemophilus influenzae*

Rabeb Dhouib^1^, Marufa Nasreen^1^, Dk Seti Maimonah Pg Othman^1^, Daniel Ellis^1^, Simon Lee^1^, Ama-Tawiah Essilfie^2^, Philip M. Hansbro^3^, Alastair G. McEwan^1^, Ulrike Kappler^1*^

^1^Australian Infectious Disease Research Centre, School of Chemistry and Molecular Biosciences, The University of Queensland, St . Lucia, Qld 4072, Australia

^2^QIMR Berghofer Medical Research Institute, 300 Herston Road, Herston QLD 4006, Australia

^3^ Centre for Inflammation, Centenary Institute and University of Technology Sydney (UTS), School of Life Sciences, Faculty of Science, Sydney, NSW 2007, Australia.

SUPPLEMENTARY FIGURES

**Table S1** Oligonucleotide primers used in this study

| **primers for pGemT-Hid*msA* kan** | |
| --- | --- |
| HI_dmsAF | CTACAAACGTTCCACTTGAAC |
| HI_dmsAR | ATGAGTAACTTTAATCAAATAAGT |
| pUC4K_PCR F | GTTGGGTAACGCCAGGGTTTTCC |
| pUC4K_PCR_R | TCCGGCTCGTATGTTGTGTGGAA |
|  | |
| **primers for complementation plasmid, p601-Hi*dmsABCDE*** | |
| HI2019dmsAcomp_Xma_F | AAAACCCGGGTGCATCTTTATTCCAAGTGGC |
| HI2019dmsAcomp_Xma_R | AAAACCCGGGCTTTCACAAGGGCGTGTTTTA |
|  | |
| **RT-QPCR primers** | |
| HI-RT_16SF | CGAAGGCAGCCCCTTGGG |
| HI-RT_16SR | CTCTAAGCCCAATCCCCAAAT |
| RT_mtsZF | AACAAACGGGTTACCACCTGC |
| RT_mtsZR | CCATTAGCGCGTATTGCTGAT |
| RT_dmsAF | CAAGCACGAACCTGATGATCA |
| RT_dmsAR | AGTAAACTGTGGTAGCCGTTG |
|  |  |
| ***dmsABCDE* co-transcription test** | |
| Hi dmsAB F | ACACCCCGTATTATTCCGG |
| Hi dmsAB R | TGACTTCTGTGCCAAGATCT |
| Hi dmsBC F | AATATGGCACACAAGCCTCC |
| Hi dmsBC R | AACAAGCCAAGCACCGAC |
| Hi dmsCD F | TAACGTTAGTAGCTGAAGGCATC |
| Hi dmsCD R | TCAAATGAAATTTCCCATTCTTGC |
| Hi dmsDE F | TCAAGCTCTTGCATTACTTACC |
| Hi dmsDE R | TTAGGGCAAGCTGAAGCG |

**Figure S1:** S-oxide reductase activity in a Hi2019^Δ^*^mtsZ^* strain grown in the absence or presence of 10 mM DMSO. In Hi2019^Δ^*^mtsZ^* DmsABC is the only S-/N-oxide reductase present.

**Figure S2**: Comparison of metabolic properties **(A)** and osmotic stress resistance **(B)** in Hi2019^WT^ and Hi2019^Δ^*^dmsA^*. Data were collected using Phenotypic Microarray plates (PM3,4,9) and a Biolog Omnilog System. Growth under a specific condition is measured through colour changes in a redox dye which are reported as a relative absorbance by the system. Both Panels report these relative absorbance units, corrected for absorbance in control wells that only contains bacteria in growth medium where applicable.

**Figure S3:** Immune cells present in BALF of mice infected with Hi2019^WT^, Hi2019^Δ^*^dmsA^* or Hi2019^Δ^*^dmsAc^*. Samples of BALF were stained using a Giemsa Stain and cell enumerated following staining. **Top:** Total immune cells, **Middle:** Neutrophils, **Bottom:** Macrophages Statistical testing used one-Way ANOVA (post-hoc test: Dunnett), * p<0.05, ** p<0.01, *** p<0.001, **** p<0.0001. Each datapoint represents one infected mouse with n=6 mice/treatment group.
